# N-acetyl-l-leucine lowers pS129-synuclein and improves synaptic function in models of Parkinson’s disease

**DOI:** 10.21203/rs.3.rs-6298077/v1

**Published:** 2025-04-09

**Authors:** Pingping Song, Rossella Franchini, Chuyu Chen, Bryan Duong, Yi-Zhi Wang, Jeffrey Savas, Loukia Parisiadou, Dimitri Krainc

**Affiliations:** Northwestern University Feinberg School of Medicine; Department of Neuroscience, Biomedicine and Movement Sciences, University of Verona; Department of Pharmacology, Feinberg School of Medicine, Northwestern University; Northwestern University Feinberg School of Medicine; Northwestern University; Department of Neurology, Feinberg School of Medicine, Northwestern University, Chicago, IL; Northwestern University; Department of Neurology Northwestern University Feinberg School of Medicine

## Abstract

N-acetyl-L-leucine (NALL), a derivative of the branched-chain amino acid leucine, has shown therapeutic potential in neurodegenerative diseases, including in prodromal stages of Parkinson’s disease (PD). However, the mechanism of its protective effects has been largely unknown. Using discovery-based proteomics, we found that treatment with NALL led to upregulation of lysosomal, mitochondrial, and synaptic proteins in PD patient-derived dopaminergic neurons. NALL reduced levels of pathological pS129-alpha-synuclein in dopaminergic neurons from patients harboring GBA1 or LRRK2 mutations. This decrease in pS129-syn was dependent on serine protease HTRA1 that was induced by NALL treatment of dopaminergic neurons. NALL also upregulated expression of wild-type parkin in both GBA1 and LRRK2 mutant neurons, leading to an increase in functional dopamine transporter and synaptic membrane-associated synaptojanin-1, suggesting improved synaptic function. Furthermore, NALL treatment of mutant LRRK2^R1441C^ knock-in mice led to decreased pS129-alpha-synuclein, increased parkin and improved dopamine-dependent motor learning deficits. These findings highlight the therapeutic potential of NALL in PD by its protective effects on α-synuclein pathology and synaptic function in vulnerable dopaminergic neurons.

## Introduction

Parkinson’s disease (PD) affects 1–2% of the population and is characterized by resting tremor, rigidity, and bradykinesia. These motor symptoms of PD are primarily due to the progressive loss of dopaminergic neurons in the substantia nigra pars compacta (SNc)^1,2^ The pathogenesis of PD involves complex interactions between genetic and environmental factors. Among the genetic factors, mutations in the genes encoding leucine-rich repeat kinase 2 (LRRK2), glucocerebrosidase (GBA), and parkin all have been identified as significant contributors that affect mitochondrial, lysosomal, and synaptic pathways^3^.

A key pathological feature of PD is the age-dependent intracellular aggregation of α-synuclein (α-syn) in spherical cytoplasmic inclusions, termed Lewy bodies, which are also observed in neuronal processes as Lewy neurites^4^. α-Syn is thought to play a central role in the pathobiology of both familial and sporadic PD. Several posttranslational modifications to α-Syn are known to occur in PD. Among them is phosphorylation of α-Syn at Ser129 (pS129-syn), a modification that plays a critical role in PD pathogenesis^5,6^. pS129-syn has been reported to enhance α-Syn toxicity both *in vivo* and *in vitro,* possibly by increasing the formation of α-Syn aggregates^6,7^. Several current studies seek to find drugs that lower the accumulation and aggregation of α-Syn in the brains of PD patients^8,9 10^.

N-acetyl-L-Leucine (NALL) is the L-enantiomer of N-acetyl-DL-leucine (ADLL), a derivative of the branched-chain amino acid leucine. ADLL has been used as a treatment for acute vertigo and vertiginous symptoms (Tanganil^®^) for many years with an excellent safety profile. More recently, it has been shown that acetyl-leucine is beneficial in patients with lysosomal storage disorders Niemann-Pick Disease Type C and GM2 gangliosidoses^11–13^. In addition, ADLL has shown promising effects in patients with cerebellar ataxia^14–16^, restless legs syndrome^17^, and more recently REM Sleep Behavior Disorder^18^ that is considered a prodrome of PD. However, the mechanism responsible for these protective effects of NALL is largely unknown.

In this study, we found that NALL promoted the degradation of pS129-syn in human iPSC-derived dopaminergic neurons with GBA1 mutations. Moreover, NALL increased expression of parkin and synaptic vesicle recycling, as well as glycosylated functional dopamine transporter (DAT). Importantly, decreased pS129-syn and increased parkin upon NALL treatment were validated in LRRK2 mutant human dopaminergic neurons and LRRK2^R1441C^ mutant mice. NALL treatment of LRRK2 mutant mice restored their behavior deficits, further pointing to the potential beneficial effects of NALL in PD.

## Results

### NALL leads to decreased pS129-syn in human dopaminergic neurons with GBA1 mutations

**Figure 1:**
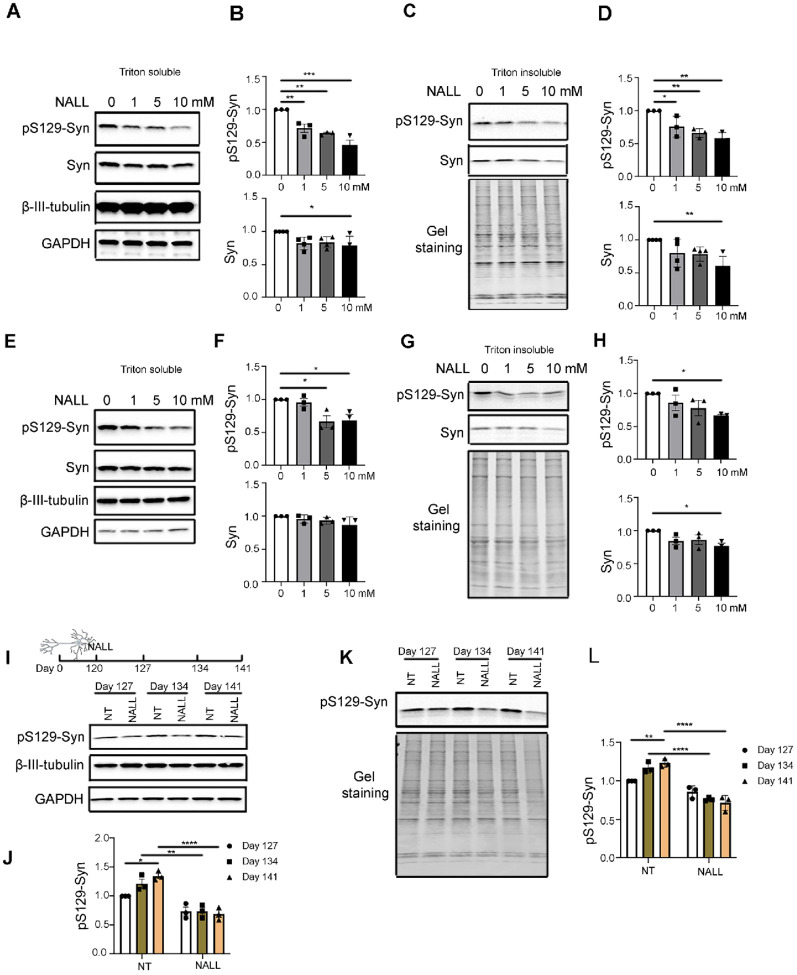
NALL leads to decreased pS129-syn in human dopaminergic neurons with GBA1 mutations **(A)** Western blot showing levels of pS129-syn and total a-Syn (Syn) upon different concentrations of NALL in Triton-soluble faction of GBA1 L444P mutant dopaminergic neurons. β-III-Tubulin and GAPDH were used as loading controls. **(B)** Quantification of the fold change of decreased pS129-syn (top) and total α-Syn (Syn)(bottom) upon NALL treatment in A. Protein levels normalized toβ-III-Tubulin (n = 3 or 4 independent experiments). **(C)** Western blot of pS129-syn and total a-Syn (Syn) upon different concentrations of NALL in Triton-insoluble faction of GBA1 L444P mutant dopaminergic neurons. Gel staining was used as a loading control. **(D)** Quantification of the fold change of decreased pS129-syn (top) and total a-Syn (Syn)(bottom) upon NALL treatment in B. Protein level was normalized with β-III-Tubulin (n = 3 or 4 independent experiments). **(E)** Western blot of pS129-syn and total a-Syn (Syn) upon different concentrations of NALL treatment in Triton-soluble faction of GBA1 N370S mutant dopaminergic neurons. β-III-Tubulin and GAPDH were used as loading controls. **(F)** Quantification of the fold change of pS129-syn (top) and total α-Syn (Syn)(bottom) upon NALL treatment in E. The protein level was normalized with β-III-Tubulin (n = 3 independent experiments). (G) Western blot of pS129-syn and total a-Syn (Syn) upon NALL treatment in Triton-insoluble faction of GBA1 N370S mutant dopaminergic neurons. Gel staining was used as a loading control. **(H)** Quantification of the fold change of pS129-syn (top) and total a-Syn (Syn)(bottom) upon NALL treatment in G. Protein level was normalized with total protein (n = 3 independent experiments). **(I)** (Top) Schematic illustration of the NALL treatment on dopaminergic neurons. GBA1 L444P mutant neurons were grown for 120 days and treated with or without 10 mM of NALL for another 7, 14, or 21 days. Cells were collected on days 127, 134, or 141. (Bottom) Western blot showing the protein level of pS129-syn with (NALL) or without (NT) NALL treatment in Triton-soluble faction. β-III-Tubulin and GAPDH were used as loading controls. **(J)** Quantification of the fold change of decreased pS129-syn in I. The protein level was normalized with β-III-Tubulin (n = 3 independent experiments). **(K)** Western blot of pS129-syn with (NALL) or without (NT) NALL treatment in Triton-insoluble faction of GBA1 L444P mutant dopaminergic neurons. Gel staining was used as a loading control. **(L)** Quantification of the fold change of pS129-syn in K. Protein level was normalized with total protein (n = 3 independent experiments). All data are represented as mean ± SEM, *p<.05, **p<.01 and ***p<.005 and ****p<.0001.

Since NALL exhibited protective effects in lysosomal disorders Niemann-Pick Disease Type C and GM2 gangliosidoses^11–13^, we hypothesized that NALL might be beneficial to PD patients with lysosomal dysfunction. To test this hypothesis, iPSC-derived dopaminergic neurons with GBA1 mutation (L444P) were treated with several concentrations of NALL on day 120 for 4 weeks. We observed a significant decrease of pS129-syn in a dose-dependent manner in both triton soluble and insoluble fractions (Fig. 1A–D), whereas total α-Syn levels were decreased with 10 mM NALL treatment. This finding was replicated in another GBA1 mutant (N370S) line where we also observed a decrease in pS129 and total syn upon NALL treatment (Fig. 1E–H). We found that phosphorylated a-Syn (pS129-syn) accumulated in both triton soluble and insoluble fractions from mutant GBA1 neurons cultured for 127 to 141 days. Upon treatment with NALL for 14 and 21 days, pS129-syn was reduced in mutant neurons (Fig. 1I–L), further suggesting that NALL treatment prevents the accumulation of a-Syn in GBA1 neurons.

### Decreased pS129-syn upon NALL treatment is mediated by HTRA1

**Figure 2:**
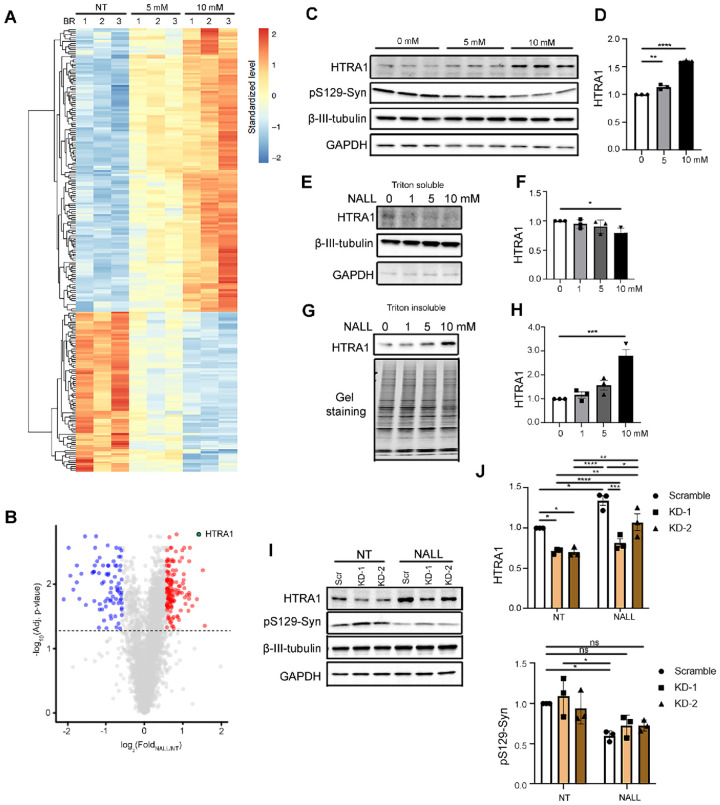
The effect of NALL on pS129-syn was mediated through increasing HTRA1. **(A)** Heatmap summarizing protein levels significantly altered (adj. *p*-value < 0.05) by NALL treatments in GBA1 L444P mutant neurons. Each condition includes three biological replicates (BR). Red and blue reflect increased and decreased, respectively. (N =3 replicates). **(B)** Volcano plot summarizing protein level alterations induced by 10 mM NALL treatment compared to non-treated (NT) samples (n = 3). Proteins significantly altered (ratio NALL/NT > 1.5 and adjusted *p*-value < 0.05) are highlighted in blue, red, or green. The green dot indicates HTRA1. The dashed line represents an adjusted *p*-value threshold of 0.05 (one-way ANOVA, Benjamini–Hochberg correction). **(C)** Western blot of HTRA1 and pS129-syn upon different concentrations of NALL treatment in total lysates of GBA1 L444P mutant dopaminergic neurons. β-III-Tubulin and GAPDH were used as loading controls. **(D)** Quantification of the fold change of increased HTRA1 upon NALL treatment in C. The protein level was normalized with β-III-Tubulin (n = 3 independent experiments). **(E)** Western blot of HTRA1 upon NALL treatment in Triton-soluble faction of GBA1 L444P mutant dopaminergic neurons. β-III-Tubulin and GAPDH were used as a loading control. **(F)** Quantification of the fold change of decreased HTRA1 upon NALL treatment in E. The protein level was normalized with β-III-Tubulin (n = 3 independent experiments). **(G)** Western blot of HTRA1 upon NALL treatment in Triton-insoluble faction of GBA1 L444P mutant dopaminergic neurons. Gel staining was used as a loading control. **(H)** Quantification of the fold change of increased HTRA1 upon NALL treatment in G. The protein level was normalized with total protein (n = 3 independent experiments). **(I)** Western blot of HTRA1 and pS129-syn with (NALL) or without (NT) NALL treatment upon HTRA1 knockdown (KD-1 and KD-2) in GBA1 L444P mutant dopaminergic neurons. β-III-Tubulin and GAPDH were used as a loading control. (J) Quantification of the fold change of HTRA1 (top) and pS129-syn (bottom) with or without NALL treatment in I. The protein level was normalized with β-III-Tubulin (n = 3 independent experiments). All data are represented as mean ± SEM, ns: not significant, *p<.05, **p<.01, ***p<.005 and ****p<.0001.

To better understand the underlying mechanisms of NALL treatment, we performed discovery-based proteomic analysis in iPS-derived dopaminergic neurons. This analysis revealed 896 proteins with significantly elevated levels and 283 proteins with decreased levels (Table S1). The proteins that were increased by at least 1.5-fold are involved in mitochondrial, lysosomal, and synaptic pathways whereas proteins that were decreased are involved in cytoskeletal function (Fig. 2A, Fig S1, Table S1, and S2). One of the top candidates was HTRA1 (Fig. 2B), an ATP-independent PDZ serine protease that cleaves various proteins such as fibronectin and amyloid precursor protein. It has been reported that HTRA1 can disaggregate a-Syn amyloid fibrils in vitro and prevent the accumulation of Lewy Body-like inclusions comprised of hyperphosphorylated a-Syn in mice^19^. Therefore, we hypothesized that increased HTRA1 might explain the effects of NALL on pS129-syn. We observed significantly elevated fold change of HTRA1 levels upon NALL treatment compared to the non-treated group (Fig. 2C and D), consistent with the proteomic data (Fig. 2B). Consistent with a previous finding that HTRA1 prominently partitions to the insoluble fraction when incubated without a-Syn, and soluble fraction in the presence of a-Syn in vitro^19^, we found a significant decrease of HTRA1 in the Triton soluble fraction but an increase in the Triton insoluble fraction with NALL treatment (Fig. 2E–H). Next, to test if HTRA1 indeed mediated NALL-induced lowering of pS129-syn, two different shRNA lentiviral constructs were used to knockdown HTRA1 (KD-1 and KD-2) in both non-treated (NT) and treated (NALL) conditions (Fig. 2I and J top panel). While HTRA1 was significantly higher and pS129-syn lower in the scramble control group, there was no significant decrease of pS129-syn upon NALL treatment when HTRA1 was knocked down (Fig. 2I and J bottom). These results suggested that NALL-mediated decrease in pS129-syn in mutant GBA1 neurons was at least in part mediated by HTRA1.

### NALL increases functional dopamine transporter and synaptic function through Parkin

**Figure 3:**
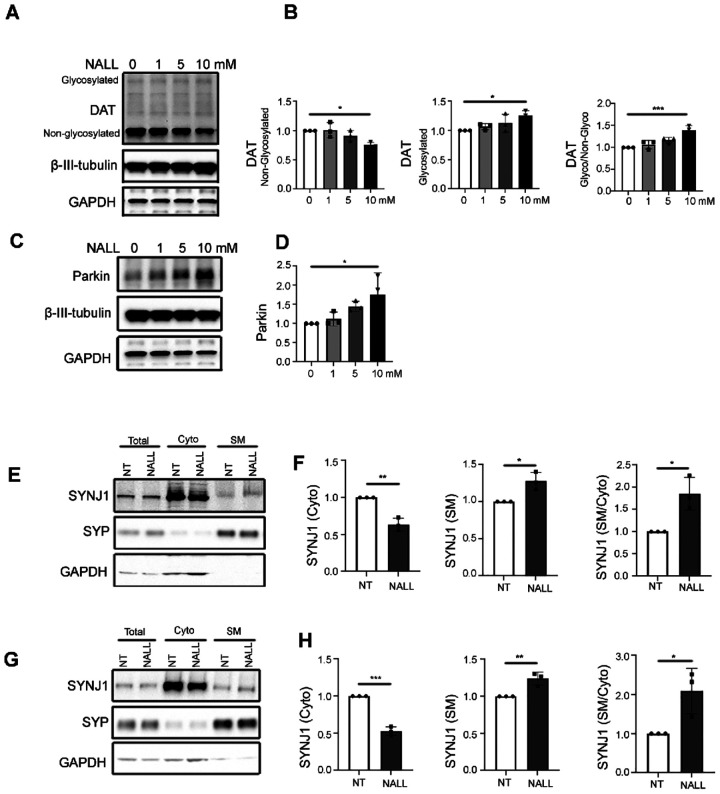
NALL increases functional dopamine transporter and synaptic function through Parkin **(A)** Western blot of glycosylated and non-glycosylated DAT upon different concentrations of NALL treatment in GBA1 L444P mutant dopaminergic neurons. β-III-Tubulin and GAPDH were used as a loading control. **(B)** Quantification of the fold change of non-glycosylated DAT (left), glycosylated DAT (middle), and the ratio of glycosylated versus non-glycosylated DAT (right) upon NALL treatment in A. Protein level was normalized with β-III-Tubulin (n = 3 independent experiments). **(C)** Western blot of parkin upon different concentrations of NALL treatment in GBA1 L444P mutant dopaminergic neurons. β-III-Tubulin and GAPDH were used as a loading control. **(D)** Quantification of the fold change of parkin upon NALL treatment in C. Protein level was normalized with β-III-Tubulin (n = 3 independent experiments). **(E)** SYNJ1 is decreased in the cytosolic fraction (Cyto) and increased in the synaptic membrane fraction (SM) upon NALL treatment compared to non-treated (NT) dopaminergic neurons carrying GAB L444P mutation. Fractions were obtained from original total cell homogenate (Total). Synaptophysin (SYP) was used as a synaptic marker. GAPDH was used as a cytosolic marker. **(F)** Quantification of the fold change of cytosolic SYNJ1, normalized with GAPDH (left), synaptic membrane fraction of SYNJ1, normalized with SYP (middle), and the ratio of synaptic membrane (SM) versus cytosolic (Cyto) SYNJ1 (right) upon NALL treatment in E (n = 3 independent experiments). **(G)** SYNJ1 is decreased in the cytosolic fraction (Cyto) and increased in the synaptic membrane fraction (SM) upon NALL treatment compared to non-treated (NT) dopaminergic neurons carrying GAB N370S mutation. Fractions were obtained from original total cell homogenate (Total). Synaptophysin (SYP) was used as a synaptic marker. GAPDH was used as a cytosolic marker. **(H)** Quantification of the fold change of cytosolic SYNJ1, normalized with GAPDH (left), synaptic membrane fraction of SYNJ1, normalized with SYP (middle), and the ratio of synaptic membrane (SM) versus cytosolic (Cyto) SYNJ1 (right) upon NALL treatment in G (n = 3 independent experiments). All data are represented as mean ± SEM, *p<.05, **p<.01 and ***p<.005.

Recent findings suggest that ADLL improved the DAT-SPECT binding ratios in patients with REM sleep behavior disorder^18^. Consistent with this finding, we found that non-glycosylated DAT was decreased (Fig. 3A and B left), whereas glycosylated DAT increased in a dose-dependent manner upon NALL treatment (Fig. 3A and B middle). As the glycosylated DAT is cell surface expressed DAT^20,21^, the increased ratio of glycosylated versus non-glycosylated DAT (Fig. 3B right) suggested an increase in functional DAT. It has been reported that parkin enhances cell surface expression of DAT by ubiquitinating and degrading non-glycosylated misfolded DAT, which affects the oligomerization and surface expression of native DAT molecules^21^. To test whether the effects of NALL on DAT might be mediated by parkin, we examined the parkin levels in GBA1 mutant dopaminergic neurons. Interestingly, we found a dose-dependent increase in parkin upon treatment with NALL (Fig. 3C and D), suggesting that increased parkin might account for the increased cell surface or functional DAT. Our previous data in dopaminergic neurons showed that parkin facilitates synaptic vesicle recycling through ubiquitination-mediated membrane recruitment of synaptojanin-1 (SYNJ1), which promotes clathrin uncoating during synaptic vesicle recycling pathway^22^. Interestingly, we observed a significant decrease in cytosolic SYNJ1 but an increase of synaptic membrane-associated SYNJ1 upon NALL treatment in both GBA1 L444P (Fig. 3E and F) and N370S (Fig. 3G and H) mutant neurons, suggesting increased synaptic vesicle endocytosis pathway upon NALL treatment. These results suggest that NALL-mediated increase in parkin protein promotes efficient synaptic vesicle recycling in mutant GBA1 neurons.

### NALL leads to decreased pS129-syn and increased parkin in mutant human and mouse LRRK2 dopaminergic neurons

**Figure 4:**
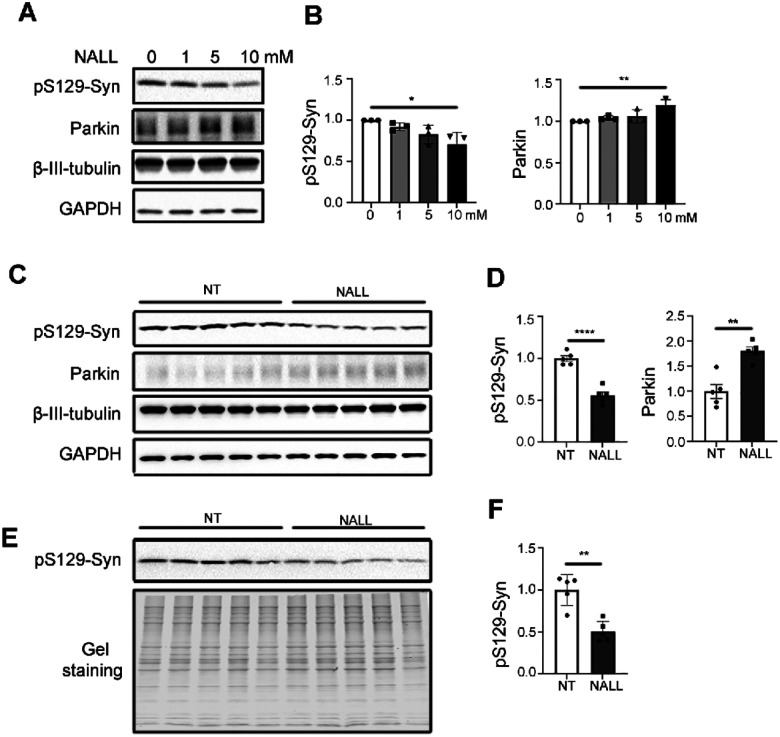
NALL leads to decreased pS129-syn but increased parkin in LRRK2 mutant dopaminergic neurons in both human and mice. **(A)** Western blot of pS129-syn and parkin upon different concentrations of NALL treatment in LRRK2 R1441C mutant dopaminergic neurons. β-III-Tubulin and GAPDH were used as a loading control. **(B)** Quantification of the fold change of decreased pS129-syn (left) and parkin (right) upon NALL treatment in A. Protein level was normalized with β-III-Tubulin (n = 3 independent experiments). **(C)** Western blot of pS129-syn and parkin with (NALL) or without (NT) NALL treatment in Triton-soluble faction of LRRK2^R1441C^ mice substantial nigra. β-III-Tubulin and GAPDH were used as a loading control. **(D)** Quantification of the fold change of decreased pS129-syn (left) and increased parkin upon NALL treatment in C. Protein level was normalized with β-III-Tubulin (n = 3 independent experiments). **(E)** Western blot of pS129-syn with (NALL) or without (NT) NALL treatment in Triton-insoluble faction of LRRK2^R1441C^ mice substantial nigra. Gel staining was used as a loading control. **(F)** Quantification of the fold change of decreased pS129-syn upon NALL treatment in E. Protein level was normalized with total protein (n = 3 independent experiments). All data are represented as mean ± SEM, *p<.05, **p<.01, ***p<.005 and ****p<.0001.

Our data so far suggested that NALL could decrease pathogenic pS129-syn level through HTRA1 and improve synaptic function through parkin in mutant GBA1 dopaminergic neurons. To understand if NALL may be beneficial in other forms of PD, we examined NALL in patient-derived dopaminergic neurons carrying LRRK2 R1441C mutation. Like the findings in mutant GBA1 neurons, we observed decreased pS129-syn and increased parkin in mutant LRRK2 neurons upon NALL treatment for 2 weeks (Fig. 4A and B). In addition, we found a significant decrease of pS129-syn in both Triton-soluble (Fig. 4C and D) and Triton-insoluble fraction (Fig. 4E and F) in LRRK2^R1441C^ mice^23^ treated with NALL. Furthermore, we also observed a significant increase of parkin protein in LRRK2 mice upon NALL treatment (Fig. 4C and D), suggesting that NALL might be beneficial in LRRK2-linked PD.

### NALL improves dopamine-dependent motor learning in LRRK2 mice

Since NALL treatment exhibited similar effects on a-Syn and parkin in LRRK2 patient derived neurons and mice, we examined whether NALL could improve the behavioral alterations in mutant LRRK2^R1441C^ knock-in mice. Earlier studies showed that the loss of dopamine antagonism during the learning of a motor skill can hinder future motor performance even after the blockade has ended^24,25^. Considering the significance of dopamine’s role in motor learning and the decrease in dopamine release in the dorsal striatum of LRRK2^R1441C^ mice^23^, we explored how dopamine antagonism affects striatal motor learning in these mice, using a two-stage accelerating rotarod paradigm, during which we applied D1 and D2 receptor antagonism in the first phase^23^. After a 72-hour break, the performance of the mice was assessed in the accelerating rotarod by measuring their latency to fall. We showed that wild-type LRRK2^wt^ but not mutant LRRK2^R1441C^ mice gradually improved their performance during the antagonism-free phase, indicating that the R1441C mutation disrupts dopamine-mediated motor learning in the striatum^23^.

**Figure 5:**
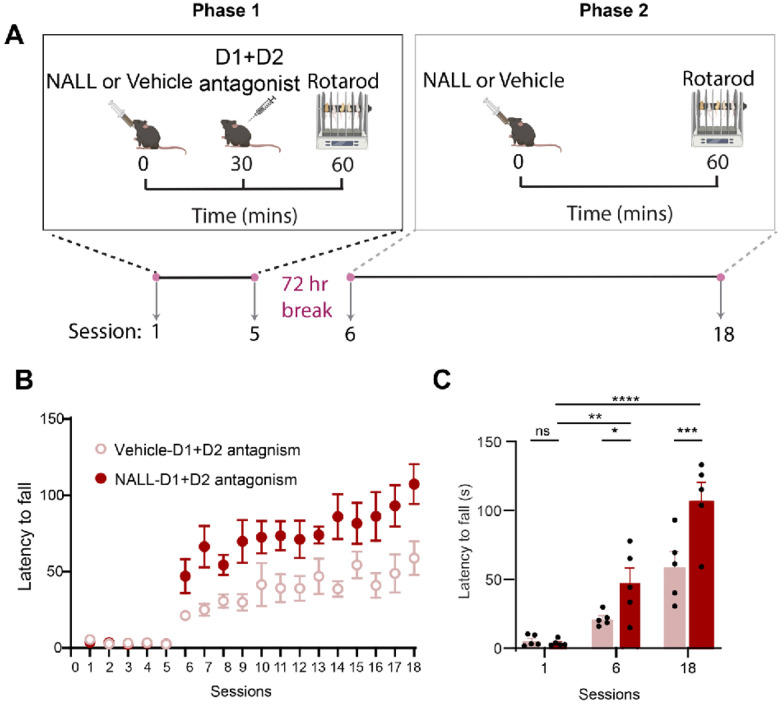
NALL improved dopamine-dependent motor learning impairments in LRRK2^r1441C^ mice. **A.** Schematic of the rotarod training paradigm. Mice were evaluated over a total of 18 daily sessions, with each session consisting of five trials. LRRK2 ^R1441C^ mice were administered either NALL or a vehicle control 30 minutes before receiving either saline or a cocktail of dopamine D1 receptor antagonists (SCH23390) and D2 receptor antagonists (eticlopride), both at a dosage of 1 mg/kg. Thirty minutes after the cocktail administration, the mice were trained on an accelerated rotarod for five consecutive days. After a 72-hour break, the mice were returned to the rotarod for an additional 13 days, with either NALL or vehicle administered 60 minutes before the accelerated rotarod test. **B** Improved performance of LRRK2 ^R1441C^ mice received NALL ( treatment x session interaction < p=0.011) **C.** Summarized data of the average latency in the session 1, session 6 and session 18 in the B. Data are represented as mean±SEM. Asterisks in C show statistical significance for Sidak’s multiple comparison tests after two-way ANOVA; *p < .05, **p< .01, ***p < .001, ****p < .0001. n=5 mice/treatment.

To test potential beneficial effects of NALL on motor performance of mutant LRRK2^R1441C^ mice, NALL was administered to LRRK2^R1441C^ mice throughout the task (Fig. 5). The treated LRRK2^R1441C^ mice showed a marked improvement compared to vehicle-treated mice across the second phase of the Rotarod protocol (treatment x session interaction < p value = .011, 2-way ANOVA). Next, we compared the performance of mutant LRRK2^R1441C^ mice across selective task sessions. As expected, the dopamine antagonist impacted the performance at the first stage of the task; no difference was found (session 1) across treatments. However, at both sessions 6 and 18 of phase II, NALL improved LRRK2 mice performance compared to vehicle-treated controls (p value = .04 for session 6 and p value = .0004 for session 18; Sidak’s multiple comparison tests after two-way ANOVA). These data suggest that NALL improves dopamine-dependent motor learning of mutant LRRK2^R1441C^ mice.

## Discussion

Our patient-derived dopaminergic neuron data demonstrated that NALL decreased pS129-syn in GBA1 and LRRK2 mutant neurons. In GBA1 L444P neurons, we also observed a decrease in total α-Syn after 4 weeks of NALL treatment. To elucidate the mechanism of NALL-mediated effects on α-Syn, we employed discovery-based proteomics that identified an increase in HTRA1 protein in human dopaminergic neurons treated with NALL. It has been previously reported that HTRA1 protects against α-Syn aggregation and hyperphosphorylation^19^. In line with this finding, we found that the decrease of pS129-syn in NALL treated neurons was dependent on the expression of HTRA1, suggesting that targeting HTRA1 activation could be a novel strategy for counteracting α-Syn pathology. Since prior studies with purified HTRA1 protein demonstrated similar effects with other aggregation-prone proteins such as FUS, TDP-43^19^ and fibrillar tau^26,27^, it would be of interest to examine whether NALL treatment exhibited protective effects in models of amyotrophic lateral sclerosis, frontotemporal dementia, and Alzheimer’s Disease.

A recent study found that acetyl-leucine reversed the loss of striatal dopamine-transporter binding in the nigrostriatal system of patients with prodromal PD^18^. However, the mechanism of these effects has been unexplored. Our results demonstrate that NALL enhanced synaptic function by increasing parkin levels and stabilizing functional DAT in patient-derived GBA1 and LRRK2 dopaminergic neurons. This aligns with previous studies indicating that parkin facilitates DAT surface expression by ubiquitinating and degrading misfolded, non-glycosylated DAT^21^. Emerging evidence suggests that parkin plays a crucial role in synaptic function, particularly in the regulation of synaptic vesicle recycling^22,28^. Neuronal activity-dependent activation of parkin in human dopaminergic neurons leads to ubiquitination of SYNJ1, facilitating its interaction with endophilin A1 and promoting synaptic vesicle recycling^22^. In agreement with these findings, NALL-mediated increase in parkin expression enhanced the localization of SYNJ1 at synaptic membrane, suggesting that NALL promotes efficient synaptic endocytosis and dopamine neurotransmission. This notion is supported by our findings in LRRK2^R1441C^ knock-in mice that exhibit impaired dopamine-dependent motor learning^23^, indicating striatal dopamine signaling dysfunction. Treatment of the LRRK2 ^R1441C^ mice with NALL led to upregulation of parkin in nigral dopaminergic neurons and improvements in their motor learning performance. Given the known effects of parkin^22^, these findings suggest that NALL-mediated parkin upregulation may help restore synaptic vesicle dynamics^29,30^ and DAT function in LRRK2 mutant neurons^31,32^.

The LRRK2^R1441C^ knock in mice^23,33,34^ serve as a valuable in vivo model for studying the early stages of PD as they present dopamine-related phenotypes but not overt dopaminergic cell loss. Changes in the dopamine-dependent striatal motor learning restored with NALL likely reflect dysfunctions in dopamine-associated circuits present in asymptomatic LRRK2 mutation carriers^35,36^. A recent work focusing on a progressive PD model provides additional evidence linking dopamine-dependent motor learning deficits with early (synaptic) dopamine neuron dysfunctions during PD progression^37^. This study found that decreased dopamine release from dopaminergic axons results in striatal motor learning deficits in the absence of PD motor impairments, which are only observed upon a significant loss of dopamine neurons. These findings further support the potential neuroprotective effects of NALL that have been reported in the clinical setting^18^. In addition, we found a reduction of pS129-syn in human dopaminergic neurons carrying LRRK2 mutation and in LRRK2 mutant mice, further supporting the role of NALL in enhancing α-Syn clearance mechanisms.

Our proteomic results indicate that NALL treatment of human dopaminergic neurons primarily increased levels of mitochondrial, synaptic and lysosomal proteins. While there could be many reasons underlying this observation, it is also possible that NALL-mediated elevation of parkin contributed to these proteomic changes in treated neurons. It has been well-established that parkin plays an important role in the mitochondrial quality control pathway^38 39–41^, as well as in lysosomal function by regulating direct contacts and transfer of amino acids between lysosomes and mitochondria^42^. It is also possible that high levels of intracellular leucine affect mTOR function^43^ and autophagy^44,45^ that in turn impact mitochondrial and lysosomal function. Further mechanistic studies will be required to address these questions.

Together, our findings support NALL as a promising therapeutic candidate for PD, by targeting α-synuclein clearance, synaptic dysfunction, and dopaminergic deficits.

## Methods

### Ethics statement

All research complied with regulatory committees for research safety (Safety Protocol ID: BIO20230025). All mouse experiments were performed in compliance with Northwestern University Animal Care and Use Committee guidelines.

#### Cells and animals

Human iPSC GBA1 L444P line was a gift from Thomas Gasser^46^; GBA1 N370S line and LRRK2 R1441C line were from the Northwestern University Biorepository. 3 month old C57BL/6 (Jax 000664, RRID:IMSR_JAX:000664) and homozygous LRRK2 ^R1441C^ knock-in mice ( RRID:IMSR_JAX:009346) were group-housed on a standard 12/12 hr light/dark cycle with standard feeding. Littermates were randomly assigned to the experimental procedures. Both males and females were used.

##### iPSC culture and neuronal differentiation:

Human iPS cells were cultured and maintained as described previously^47^. Directed differentiation toward dopaminergic neurons was conducted as described previously^47–50^. Briefly, undifferentiated iPS cells were plated onto Cultrex-coated (BD Bioscience) 6-well plates in mTeSR media (Stemcell technologies). Differentiation was started by adding knock-out serum replacement medium (Invitrogen) containing Noggin (R&D Systems) and SB431542 (Tocris Bioscience). To help control neuralization variability, cells were passaged by chunking manually (en bloc: size of 1–2mm) and plated onto 10 cm dishes precoated with poly-D-lysine (Sigma) and laminin (Roche) on day 13. Differentiation into dopaminergic neurons was conducted by adding Neuralization growth factors. All the experiments were done between day 80 to day 150. For N-Acetyl-l-Leucine (NALL) (Sigma, 441511) treatment, NALL was dissolved in DMSO at 1M for stock and diluted into cell culture media to get final concentrations as indicated in the figures. Neurons were treated with different concentrations of NALL twice a week for indicated days.

###### Mouse motor learning

NALL was dissolved in ethanol to prepare 50 mg/ml solution, which was then diluted in water to get 10 mg/ml. 0.25 ml of NALL solution (10 mg/ml) was orally administered in mice at 100 mg/kg/day dose (2.5 mg NALL/25g mouse)^51^. Motor learning was assessed using an accelerating rotarod with 8–9 weeks old LRRK2 R1441C mice. The rotarod apparatus (Panlab) is equipped with a mouse rod (3 cm diameter) and set to 4–40 rpm acceleration over 300 s. The task consisted of eighteen daily sessions (five trials per session; intertrial-interval = 15 s, max trial duration = 300 s) divided into two phases*^2^. In the first phase—session 1–5— mice were administered either NALL or vehicles via oral gavage 30 min before an intraperitoneal (i.p.) injection of a cocktail of D1 receptor (SCH23390, Sigma D054) and D2 receptor (eticlopride, Sigma E101). Each antagonist was administered a dose of 1 mg/kg for 30 minutes. After a 72-hour break, the mice were tested over thirteen sessions without dopamine antagonists. Instead, they received either NALL or vehicle via oral gavage 60 minutes before the rotarod test.

###### HTRA1 lentivirus

Lentiviral shRNA constructs (MISSION pLKO.1-puro) for non-target control and HTRA1 were obtained from Millipore Sigma. For lentiviral packaging, HEK293FT cells were transfected with lentiviral expression constructs together with psPAX2 (Addgene, 12260) and pLP3 (Invitrogen) using X-tremeGENE HP DNA Transfection Reagent (Roche). A media change was performed the day after transfection. 48 h after transfection, virus-containing supernatant was collected and cleared by centrifuge at 500 x *g* for 10 minutes. Virus particles were concentrated using Lenti-X Concentrator (Takara Bio, 631232) following manufacturer’s instructions. The concentration of virus particles was determined by ELISA using RETRO-TEK HIV-1 p24 Antigen ELISA Kit (ZeptoMetrix, 0801111).

###### Western blot analysis

Cells were scraped in cold PBS and centrifuged at 300 x g for 5 min. Pellets were resuspended in Triton buffer (containing 1% Triton X-100) with Halt Protease and Phosphatase Inhibitor Cocktail (Thermo Fisher) and incubated on ice for 30 min followed by homogenized for 2 minutes. Lysates were cleared by centrifugation at 100,000 × *g* for 30 minutes. Protein concentration of the supernatants, as Triton soluble fraction, was measured using the Bicinchoninic Acid assay (Sigma). Pellets were resolved in 2% SDS buffer, followed by sonication and centrifugation at 150,000 × *g* for 30 minutes. The supernatant was Triton-insoluble fraction. Both Triton-soluble and insoluble fractions were denatured by heating in 4X Laemmli sample buffer (BioRad). Equal amounts of protein were loaded in 4–20% Tris-glycine gels. Proteins were transferred onto Nitrocellulose membrane using the Transblot Turbo transfer system (BioRad). Membranes were blocked with 5% milk in TBS-T (50 mM tris, pH 7.4, 150 mM NaCl, 0.1% Tween20) and incubated with primary antibody overnight at 4°C. After washing, membranes were incubated with anti-rabbit or anti-mouse secondary antibody for 1 h at room temperature. Chemiluminescence was assessed using pico or femto chemiluminescence substrates (Thermo Fisher). ChemiDoc XRS + imaging station with a 16-bit CCD camera was used for imaging. Quantification was done using ImageJ software (NIH). Antibodies used for immunoblotting include anti-pS129-syn antibody (Cell Signaling), anti-SYP antibody (Millipore), anti-a-syn antibody (Santa Cruz), anti-β-III-Tubulin antibody (BioLegend), anti-synaptojanin-1 (SYNJ1) antibody (LsBio), anti-parkin antibody (Santa Cruz), anti-HTRA1 (R& D Systems), anti-DAT antibody (Santa Cruz), and anti-GAPDH antibody (Millipore).

###### Synaptic membrane enrichment assay

Cells grown in 6 well plates were washed with cold PBS and scrapped in Syn-PER^™^ Synaptic Protein Extraction Reagent (Syn-PER) (Thermo Fisher Scientific) followed by centrifuging at 1200 xg for 10 min at 4°C. Supernatants were collected as homogenates (Total) and further centrifuged at 15,000 xg for 20 min at 4°C. The supernatants from the second spin contained cytosolic proteins (Cyto). The pellets were re-solubilized in Syn-PER and centrifuge at 70,000 × *g* for 45 min at 4°C. The supernatants from this spin were added into the Cyto fraction, and the pellets were washed with Syn-PER buffer twice and finally resuspended in Syn-PER as the synaptic membrane fraction (SM) which contained SV membrane and synaptic plasma membrane. Equal amounts of protein from each fraction were loaded and detected by western blot using indicated antibodies.

### Label-free Quantitative Proteomics:

#### Sample preparation:

iPSC-derived midbrain dopaminergic neurons treated with 0, 5, or 10 mM of NALL twice a week for 4 weeks. Cells were harvested for label-free quantitative proteomic analysis. Briefly, neurons were harvested in 1x PBS and pelleted at 3000 × *g* for 5 min at 4°C. RIPA buffer contains complete protease inhibitor cocktail was added to the pellet at a volume of 1 mL of lysis buffer per 250uL cell pellet volume. Samples were homogenized using a tip sonicator 3 × 30 sec with rests of 1 min in between. Lysates were centrifuged at 10,000 × *g* for 10 min at 4°C. Supernatant was collected for protein quantification using the BCA assay. Protein pellets were resuspended in 8 M urea (Thermo Fisher Scientific, Cat# 29700) prepared in 100 mM ammonium bicarbonate solution (Fluka, Cat# 09830). Dithiothreitol (DTT, DOT Scientific Inc, Cat# DSD11000) was applied to a final concentration of 5 mM. After incubation at RT for 20 mins, iodoacetamide (IAA, Sigma-Aldrich, Cat# I1149) was added to a final concentration of 15 mM and incubated for 20 mins at RT in the dark. Excess IAA was quenched with DTT for 15 mins. Samples were diluted with 100 mM ammonium bicarbonate solution, and digested for three hrs with Lys-C protease (1:100, Thermo Fisher Scientific, Cat# 90307_3668048707) at 37°C. Trypsin (1:100, Promega, Cat# V5280) was then added for overnight incubation at 37°C with intensive agitation (1000 rpm). The next day, reaction was quenched by adding 1% trifluoroacetic acid (TFA, Fisher Scientific, Cat# O4902–100). The samples were desalted using Peptide Desalting Spin Columns (Thermo Fisher Scientific, Cat# 89852). All samples were vacuum centrifuged to dry.

#### Tandem Mass Tag (TMT) labeling:

Our protocol was based on previously reported methods^52–54^. C18 column-desalted peptides were resuspended with 100 mM HEPES pH 8.5 and the concentrations were measured by micro BCA kit (Fisher Scientific, Cat# PI23235). For each sample, 100 μg of peptide labeled with TMT reagent (0.4 mg, dissolved in 40 μl anhydrous acetonitrile, Thermo Fisher Scientific, Cat# A44520) and made at a final concentration of 30% (v/v) acetonitrile (ACN). Following incubation at RT for 2 hrs with agitation, hydroxylamine (to a final concentration of 0.3% (v/v)) was added to quench the reaction for 15 min. TMT-tagged samples were mixed at a 1:1:1:1:1:1:1:1:1 ratio. Combined sample was vacuum centrifuged to dryness, resuspended, and subjected to Peptide Desalting Spin Columns (Thermo Fisher Scientific, Cat# 89852).

### Peptide fractionation

We used a high pH reverse-phase peptide fractionation kit (Thermo Fisher Scientific, Cat# 84868) to get eight fractions (5.0%, 10.0%, 12.5%, 15.0%, 17.5%, 20.0%, 22.5%, 25.0% and 50% of ACN in 0.1% triethylamine solution). The high pH peptide fractions were directly loaded into the autosampler for MS analysis without further desalting.

#### Tandem Mass spectrometry.

Two micrograms of each fraction or sample were auto-sampler loaded with a Thermo Vanquish Neo UHPLC system onto a PepMap Neo Trap Cartridge (Thermo Fisher Scientific, 174500; diameter, 300 μm; length, 5 mm; particle size, 5 μm; pore size, 100 Å; stationary phase, C18) coupled to a nanoViper analytical column (Thermo Fisher Scientific, 164570; diameter, 0.075 mm; length, 500 mm; particle size, 3 μm; pore size, 100 Å; stationary phase, C18) with a stainless-steel emitter tip assembled on the Nanospray Flex Ion Source with a spray voltage of 2,000 V. An Orbitrap Ascend (Thermo Fisher Scientific) was used to acquire all the MS spectral data. Buffer A contained 99.9% H2O and 0.1% formic acid, and buffer B contained 80.0% acetonitrile, 19.9% H2O with 0.1% formic acid. For each fraction, the chromatographic run was for 4 hours in total with the following profile: 0–7% for 7, 10% for 6, 25% for 160, 33% for 40, 50% for 7, 95% for 5 and again 95% for 15 mins receptively.

We used a multiNotch MS^3^-based TMT method to analyze all the TMT samples^55–57^. The scan sequence began with an MS^1^ spectrum (Orbitrap analysis, resolution 120,000, 400–1400 Th, AGC target 2×10^5^, maximum injection time 200 ms). MS^2^ analysis, ‘Top speed’ (2 s), Collision-induced dissociation (CID, quadrupole ion trap analysis, AGC 4×10^3^, NCE 35, maximum injection time 150 ms). MS^3^ analysis, top ten precursors, fragmented by HCD prior to Orbitrap analysis (NCE 55, max AGC 5×10^4^, maximum injection time 250 ms, isolation specificity 0.5 Th, resolution 60,000).

#### MS data analysis and quantification:

Protein identification/quantification and analysis were performed with Integrated Proteomics Pipeline - IP2 (Bruker, Madison, WI. http://www.integratedproteomics.com) using ProLuCID^58,59^, DTASelect2^60,61^, Census and Quantitative Analysis (For TMT-MS experiments). Spectrum raw files were extracted into MS1, MS2 and MS3 (For TMT experiments) files using RawConverter (http://fields.scripps.edu/downloads.php). The tandem mass spectra were searched against UniProt human (downloaded on 01–01-2014) protein databases^62^ and matched to sequences using the ProLuCID/SEQUEST algorithm (ProLuCID version 3.1) with 5 ppm peptide mass tolerance for precursor ions and 600 ppm for fragment ions. The search space included all fully and half-tryptic peptide candidates within the mass tolerance window with no-miscleavage constraint, assembled, and filtered with DTASelect2 through IP2. To estimate peptide probabilities and false-discovery rates (FDR) accurately, we used a target/decoy database containing the reversed sequences of all the proteins appended to the target database^62^. Each protein identified was required to have a minimum of one peptide of minimal length of six amino acid residues; however, this peptide had to be an excellent match with an FDR < 1% and at least one excellent peptide match. After the peptide/spectrum matches were filtered, we estimated that the peptide FDRs were ≤ 1% for each sample analysis. Resulting protein lists include subset proteins to allow for consideration of all possible protein forms implicated by at least two given peptides identified from the complex protein mixtures. Then, we used Census and Quantitative Analysis in IP2 for protein quantification of TMT-MS experiments and protein quantification was determined by summing all TMT report ion counts. For TMT experiments: static modification: 57.02146C for carbamidomethylation, 304.2071 for 16-plex TMT tagging; differential modifications: 304.2071 for N-terminal 16-plex TMT tagging, 42.0106 for N-terminal Acetylation. Resulting protein lists include subset proteins to allow for consideration of all possible protein isoforms implicated by at least two given peptides identified from the complex protein mixtures. Each possible protein isoforms was quantified individually^63^.

Spyder (MIT, Python 3.7, libraries, ‘pandas’, ‘numpy’, ‘scipy’, ‘statsmodels’ and ‘bioinfokit’) was used for data statistical analyses. RStudio (version, 2024.09.1 Build 394, packages, ‘tidyverse’, ‘pheatmap’) was used for data virtualization. The Database for Annotation, Visualization and Integrated Discovery (DAVID) was used for protein functional annotation analysis

### Statistical analysis

Statistical calculations were performed using GraphPad Prism 9 software. Datasets with two samples were analyzed using a two-tailed t-test. Datasets with more than two samples were analyzed using one-way ANOVA followed by Tukey’s post-hoc test.

## Data Availability

The raw mass spectrometry data presented in this study was deposited in Mass Spectrometry Interactive Virtual Environment (MassIVE) and ProteomeXchange upon acceptance.
